# Impulse dynamics of coupled synchronous neurons

**DOI:** 10.1186/1471-2202-13-S1-P102

**Published:** 2012-07-16

**Authors:** Epaminondas Rosa, Samuel Krueger

**Affiliations:** 1 Department of Physics, Illinois State University, Normal, IL 61790, USA

## 

Synchronization of neurons remains a topic of great interest due in part to practical implications of relevance to desirable as well as to undesirable states. Neural synchrony can be related, for instance, to healthy activity as in the case of the various stages of sleep [1], but can also be related to pathological processes as in the case of Parkinson’s disease [2]. Even though the enormous scientific and technological advances we have been witnessing over the past years helping us understand better the mechanisms behind neurological activities, there is still much to learn, in a context where mathematical models are potentially capable of making important contributions.

**Figure. 1:**
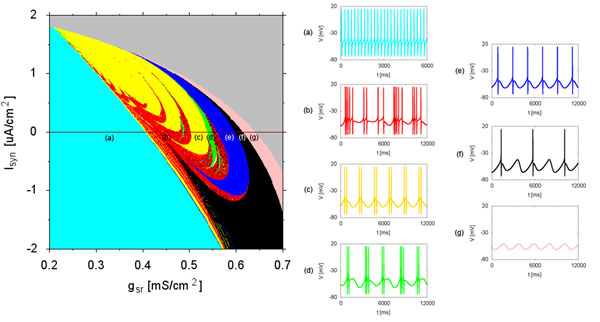
Conductivity *g_sr_vs.* synaptic current *I_sr_* color map with the corresponding firing patterns on the right-hand side.

This presentation comprises two parts. Part 1 describes a set of neuron model equations based on the Huber-Braun (HB) work which was originally intended for studying temperature sensitive neurons [3]. However, the HB equations have been proven to be applicable to a wide range of situations, including studies aimed at clarifying the mechanisms controlling the different neural firing regimes and transitions between them [4] as illustrated in Fig. 1, as well as neuropsychiatric disorders [5], to name a few. Part 2 details how these neurons can get in synchrony and shows patterns of synchronous behaviors associated with a variety of coupling values and with the firing regimes the neurons were at before being coupled. The argument will be made regarding neuropathologies such as deep depression, for example, in relation of pharmacological treatments capable of altering neuronal firing rates, therefore affecting their synchronization capability.
